# Safety and tolerability of Sacubitril/Valsartan in heart failure patient with reduced ejection fraction

**DOI:** 10.1186/s12872-023-03070-9

**Published:** 2023-03-13

**Authors:** Muhammad Nauman Khan, Najia Aslam Soomro, Khalid Naseeb, Usman Hanif Bhatti, Rubina Rauf, Iram Jehan Balouch, Ali Moazzam, Sonia Bashir, Tariq Ashraf, Musa Karim

**Affiliations:** 1 National Institute of Cardiovascular Diseases (NICVD), Karachi, Pakistan; 2 Liaquat National Hospital, Karachi, Pakistan; 3 National Institute of Cardiovascular Diseases (NICVD), Hyderabad, Pakistan; 4 Karachi Institute of Heart Diseases (KIHD), Karachi, Pakistan

**Keywords:** Sacubitril, Heart failure, Reduced ejection fraction, Safety and tolerability

## Abstract

**Background:**

Angiotensin receptor blocker and a neprilysin inhibitor (ARNI) has emerged as an innovative therapy for patients of heart failure with reduced ejection fraction (HFrEF). The purpose of this study was to assess the safety and tolerability of Sacubitril/Valsartan in patient with HFrEF in Pakistani population.

**Methods:**

This proof-of-concept, open label non-randomized clinical trial was conducted at a tertiary care cardiac center of Karachi, Pakistan. Patients with HFrEF were prescribed with Sacubitril/Valsartan and followed for 12 weeks for the assessment of safety and tolerability. Safety measures included incidence of hypotension, renal dysfunction, hyperkalemia, and angioedema.

**Results:**

Among the 120 HFrEF patients, majority were male (79.2%) with means age of 52.73 ± 12.23 years. At the end of 12 weeks, four (3.3%) patients died and eight (6.7%) dropped out of the study. In the remaining 108 patients, 80.6% (87) of the patients were tolerant to the prescribed dose. Functional class improved gradually with 75.0% (81) in class I and 24.1% (26) in class II, and only one (0.9%) patient in class III at the end of 12 weeks. Hyperkalemia remains the main safety concern with incidence rate of 21.3% (23) followed by hypotension in 19.4% (21), and renal dysfunction in 3.7% (4) of the patients.

**Conclusions:**

Sacubitril/Valsartan therapy in HFrEF patients is safe and moderately tolerated among the Pakistani population. It can be used as first line of treatment for these patients.

***Trial registration*:**

NCT05387967. Registered 24 May 2022—Retrospectively registered, https://clinicaltrials.gov/ct2/show/NCT05387967

## Background

Heart failure (HF) is a prominent cause of morbidity and death, as well as a major financial burden on healthcare systems worldwide [1–3]. The pathogenesis of HF is based on over-activation of neurohumoral mechanisms. Angiotensin-converting enzyme inhibitors (ACEIs) have been found to lower overall HF mortality by 16–40% since the release of the CONSENSUS study in 1987 and the SOLVD-Treatment trial in 1991 [4, 5]. In 2011, the EMPHASIS-HF study [6] verified and expanded the use of MRA eplerenone in patients with mild symptomatic HF. The foundations of contemporary HF treatment are these neurohumoral antagonists.

HF remains a significant source of morbidity and mortality despite various therapies aimed at neurohumoral blocking. Sacubitril/Valsartan is a novel combination drug containing an angiotensin receptor blocker (ARB) (Valsartan) and a neprilysin inhibitor (Sacubitril) (ARNI) approved by the US food and Drug Administration (FDA) and the European Medicines Agency (EMA) for the treatment of patients with HF with reduced ejection fraction (HFrEF) [7–9]. Following the release of the PARADIGM-HF study in 2014 [10], another paradigm change in HF therapy occurred. When compared to Enalapril, use of ARNI had lowered all-cause mortality by 16% and cardiovascular mortality by 20%.

The American College of Cardiology (ACC)/American Heart Association (AHA) and the European Society of Cardiology (ESC), according to this study, recently revised evidence-based recommendations for the management of HF [7–9]. Both clinical practice guidelines recommended Sacubitril/Valsartan as a class I indication in patients with persistent symptomatic HF with reduced ejection fraction (HFrEF) despite optimum therapy.

Since there is a scarcity of local evidence regarding safety and tolerability of using ARNI in HFrEF patients. Therefore, this study was conducted with an aim of to assess the safety and tolerability of Sacubitril/Valsartan, over a period of 12 weeks, in HFrEF patients coming to a tertiary care public sector hospital located in Karachi.

## Methods

This study was designed as an open label non-randomized clinical trial being conducted in the Adult Cardiology Department of National Institute of Cardiovascular Diseases (NICVD), Karachi, Pakistan during January 2021 to June 2021. The sample for this study was calculated to be n = 121, keeping the confidence level at 95% and a 7% margin of error with anticipated treatment success of 81.1% [11].

**Fig. 1:**
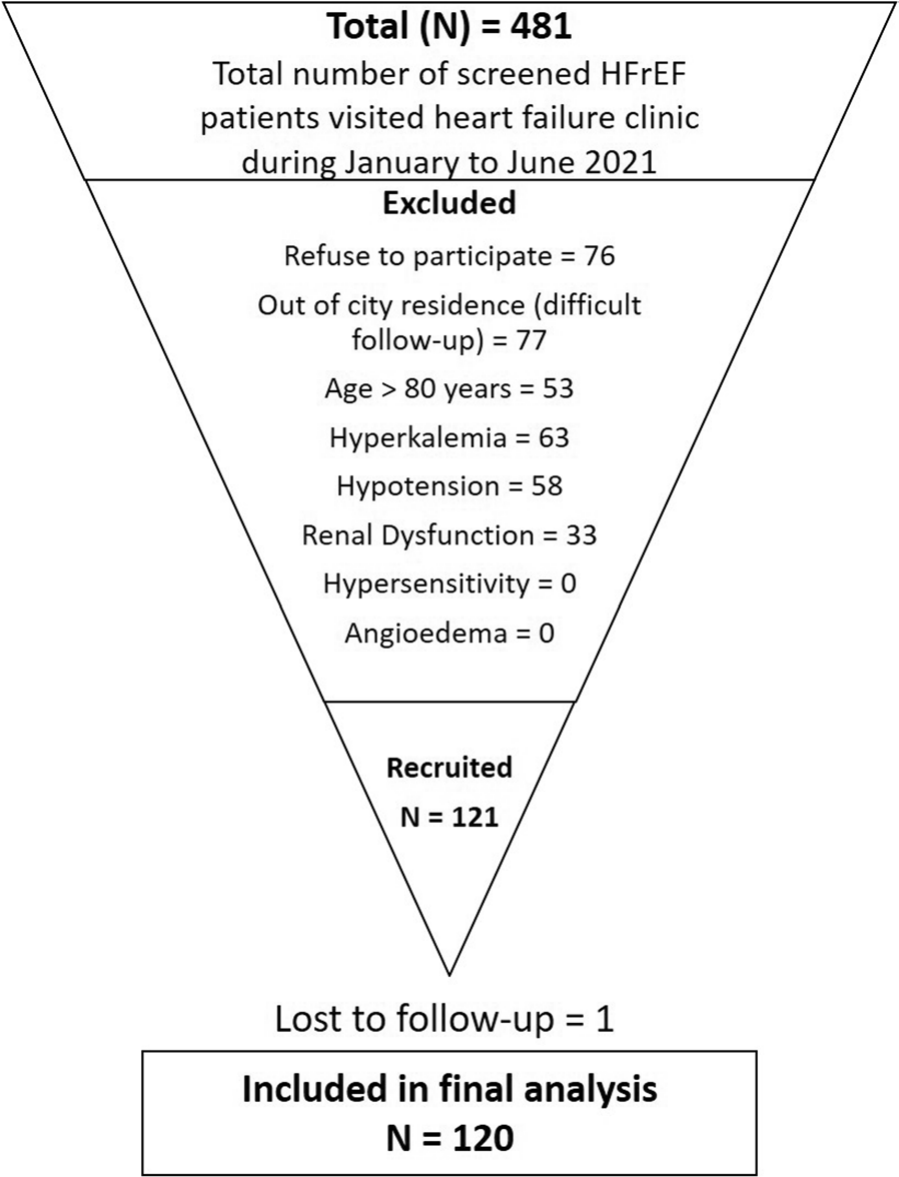
Study flow chart. *HFrEF* heart failure with reduced ejection fraction

In clinical trial was performed in accordance with the Declaration of Helsinki, study was started after obtaining approval from ethical review committee of NICVD (ERC-05/2020) and written informed consent was obtained from all the patients regarding their participation in the study and publication of data while maintaining confidentiality and anonymity. This clinical trial was registered at ClinicalTrial.gov with NCT05387967. The required number of consecutive patients meeting the inclusion criteria were recruited for this study. After obtaining written informed consent, patient’s demographic and baseline clinical characteristics were obtained. Inclusion criteria for the study are either gender, between 18 and 80 years of age, diagnosed with HFrEF with New York Heart Association (NYHA) class II-IV, and left ventricular ejection fraction (LVEF) ≤ 40%. Pre-inclusion safety parameter were patients who were stable on any dose of beta blockers, ACEI or ARB prior to enrolment in the study. Patients who refused to participate in the study or patients with hyperkalemia (baseline potassium > 5.2 mmol/L), hypotension (baseline systolic blood pressure (SBP) < 90 mmHg), renal dysfunction (baseline estimated glomerular filtration rate (eGFR) < 30 mL/min), anemia (hemoglobin level: < 13.5 g/dL in men and < 12.0 g/dL in women), and history of hypersensitivity to the active substances, Sacubitril/Valsartan, or to any of the excipients or drugs of similar chemical classes were excluded from the study (Fig. 1). In addition to the study specific exclusion criteria (mentioned above), the pre-recruitment initial screen criteria were patients with the history of prior heart failure hospitalization, on anti-coagulants, receiving Sodium/glucose cotransporter-2 inhibitors (SGLT2i) therapy, or with either CRT (Cardiac Resynchronization Therapy) or ICD (implantable cardioverter-defibrillator) device. These patients were screened out from the study considering the potential confounding role.

All the recruited patients were prescribed with Sacubitril/Valsartan at a starting dose of 50 (24/26) mg BID which was up-titrated, over the period of initial 6 weeks, to the maximum tolerated dose up to 200 (97/103) mg BID and further followed for a total of 12 weeks. A weekly telephonic follow-up was made to assess the patient’s medication adherence level and any adverse events. All the patients were kept under a close follow-up for the period of 12 weeks, the safety and tolerability outcomes were assessed.

Safety parameters included incidence of any of the following during 12 weeks of follow-up period; hypotension (SBP < 90 mmHg), renal dysfunction (eGFR < 30 mL/min), hyperkalemia (potassium > 5.2 mmol/L), and angioedema (rapid edema, or swelling, of the area beneath the skin or mucosa). Tolerability was defined as the dose tolerated by the patients which did not require down titration or discontinuation of prescribed dose during follow-up with the frequency of follow-up after 1st week, 2nd week, 4th week, 8th week, and 12th week. Functional status of all the patients was also assessed as NYHA functional class on every follow-up week. Echocardiography was performed, at baseline as well as at 12th week follow-up, and improvement in left ventricular ejection fraction, systolic, and diastolic dimension were evaluated.

All the collected information was recorded using a predefined structural proforma. Collected data were analyzed using SPSS version-21 (IBM Corp). Mean ± standard deviation (SD) were computed for the quantitative (continuous) variables, such as hemodynamic parameters, laboratory parameters, and echocardiographic parameters, and paired samples t-test was conducted to compare baseline with 12th week assessments. NYHA classification before and at the end of 12-weeks therapy was compared with the help of Chi-square test. Frequency and percentages were calculated for categorical variables such as tolerability and safety measures and Chi-square test/ Fisher's exact test were applied for the compare the results for various baseline characteristics of the patients.

## Results

### Baseline demographics

A total of 120 enrolled patients meeting eligibility criteria with successful 12 weeks follow-up were enrolled in the study and out of which four (3.3%) died during the study duration and eight (6.7%) patients discontinued medication on their own, hence excluded from the final analysis. One of these patient moved out of town and refused to visit for follow-up after first week of treatment, and remaining seven patients (5 after 1st week and 2 after 2nd week of treatment) discontinued the study medication and self-withdrawn from the study with no reported complications or symptoms. Out of the remaining 108 patients, 78.7% (85) were male and the mean age of study patients was 53.04 ± 11.89 years. Whereas, 64.8% (70) of the study population had coronary artery disease, 56.5% (61) had diabetes mellitus, and 41.7% (45) had hypertension as the comorbidity (Table 1). A total of 65.8% (79/120) were on beta blockers, 24.2% (29/120) were on diuretics, and 4.2% (5/120) patients were on digoxin.

**Table 1:** Distribution of baseline demographic characteristics of the study patients

	Total
Total (N)	120
Gender	
Male	79.2% (95)
Female	20.8% (25)
Age (years)	52.73 ± 12.23
≤ 50 years	38.3% (46)
> 50 years	61.7% (74)
Weight (kg)	70.08 ± 15.79
Risk profile	
Hypertension	44.2% (53)
Diabetes mellitus	55.8% (67)
Smoking	35% (42)
Coronary artery disease	65.8% (79)
Cerebrovascular accident (CVA)/stroke	0.0% (0)
Atrial fibrillation	9.2% (11)

### Clinical characteristics

A significant improvement (*p* = 0.010) in NYHA function class was observed after 12 weeks of therapy with a majority of patients, 75% (81), in class I at 12 weeks as against majority in class II, 71.3% (77), at baseline. Similarly, a significant improvement in left ventricular EF was also observed from 26.71 ± 5.35% at baseline to 33.36 ± 12.06% after 12-week therapy along with significant improvement in systolic dimensions (Table 2).

**Table 2:** Comparison of functional class, hemodynamics, laboratory, and echocardiographic parameters at baseline and after 12 weeks

Characteristics	Baseline	At 12th week	*p* value
Total (N)	108	108	–
Hemodynamics			
Systolic blood pressure (mmHg)	119.68 ± 25.23	112.15 ± 19.89	0.002*
Diastolic blood pressure (mmHg)	70.48 ± 16.27	66.83 ± 13.82	0.019*
Laboratory parameters			
Creatinine (mg/dL)	1.05 ± 0.32	1.05 ± 0.27	0.967
Potassium (mg/dL)	4.39 ± 0.54	4.55 ± 0.4	0.005*
eGFR (mL/min)	77.42 ± 27.71	75.89 ± 26.52	0.582
Echocardiography			
Ejection fraction (%)	26.71 ± 5.35	33.36 ± 12.06	< 0.001*
Systolic dimensions (mm)	45.83 ± 9.94	42.52 ± 11.44	0.004*
Diastolic dimensions (mm)	57.55 ± 8.7	56.71 ± 9.63	0.228
NYHA functional class			
I	0% (0)	75% (81)	0.010*
II	71.3% (77)	24.1% (26)	
III	25% (27)	0.9% (1)	
IV	3.7% (4)	0% (0)	

*eGFR* estimated glomerular filtration rate, *NYHA* New York Heart Association

*Significant at 5%

**Fig. 2:**
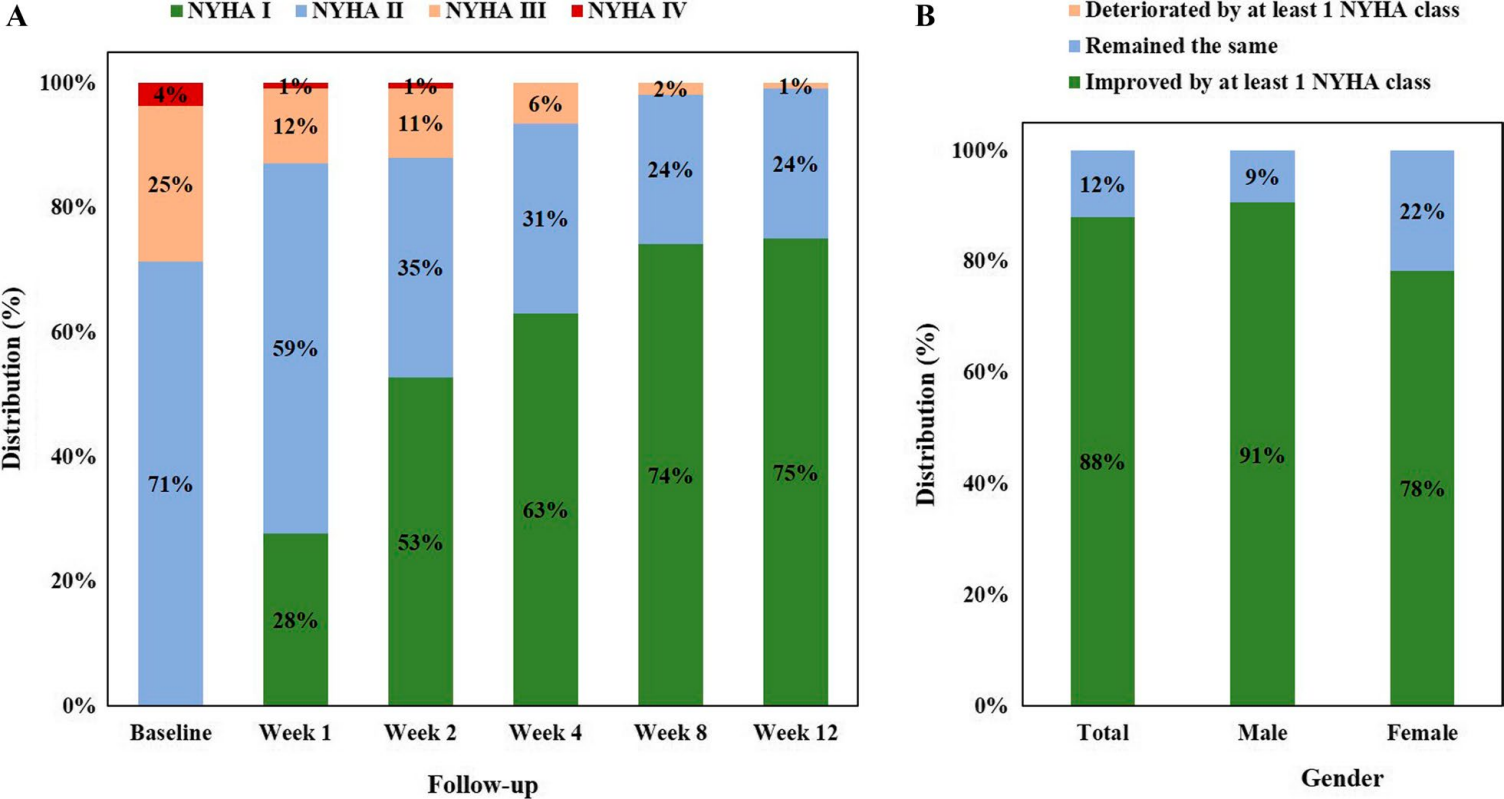
Distribution of the New York Heart Association Functional Classification on every follow-up week (**A**) and functional class improvement status by gender (**B**). *NYHA* New York Heart Association Functional Classification

The distribution of NYHA classification on every follow-up week is presented in Fig. 2A. Functional class improved by at least one NYHA class in 88% of the patients, remained the same in 12%, and did not deteriorated in any of the patient. Functional class improvement in male and female patients is provided in Fig. 2B.

### Safety

When measuring the safety of the drug in the participants, four clinical parameters were taken into consideration i.e., the occurrence of either hypotension, hyperkalemia, renal dysfunction, or angioedema among the participants. During the study duration of 12 weeks, hyperkalemia remains the main safety concern with incidence rate of 21.3% (23) followed by hypotension in 19.4% (21), and renal dysfunction in 3.7% (4) of the patients. However, none of the patient developed angioedema during this study. When assessed for various baseline characteristics, the incidence rate of hypotension was significantly higher (*p* = 0.017) among non-hypertensive patients with an incidence rate of 29.8% (14/37) as against 11.5% (7/50) among hypertensive patients. Female patients had higher incidence of renal dysfunction compared to male patients with incidence rate of 13% (3/19) vs. 1.2% (1/68), *p* = 0.030, respectively. Hyperkalemia was found to be associated with the presence of diabetes (*p* = 0.010) with the incidence rate of 33.3% (15/36) compared to 12.7% (8/51) for non-diabetic patients.

### Tolerability

A total of 80.6% (87) of the patients were tolerant to the prescribed dose with the distribution of 8% (7/87) on 50 mg BID, 28.7% (25/87) on 100 mg BID, and remaining 63.2% (55/87) on 200 mg BID. A total of 21 (19.4%) patients required either down titration or temporary discontinuing of the prescribed medication, 10 patients due to increase complained of vertigo, 5 patients got symptomatic, 3 patients due to increase in creatinine level, and 3 patients due to increase in potassium level. Tolerance was observed to be unaffected by the demographic and baseline characteristics of the patients, shown in Table 3.

**Table 3:** Comparison of tolerability and safety measures during 12-weeks of therapy by various baseline and demographic characteristics

Characteristics	Tolerability	Safety measurements		
		Hypotension	Renal dysfunction	Hyperkalemia
Gender				
Male	80% (68)	22.4% (19)	1.2% (1)	18.8% (16)
Female	82.6% (19)	8.7% (2)	13% (3)	30.4% (7)
*p* value	> 0.999	0.234	0.030*	0.256
Age				
≤ 50 years	82.5% (33)	22.5% (9)	0% (0)	17.5% (7)
> 50 years	79.4% (54)	17.6% (12)	5.9% (4)	23.5% (16)
*p* value	0.695	0.538	0.294	0.460
Hypertension				
No	78.7% (37)	29.8% (14)	2.1% (1)	21.3% (10)
Yes	82% (50)	11.5% (7)	4.9% (3)	21.3% (13)
*p* value	0.673	0.017*	0.631	0.996
Diabetes mellitus				
No	81% (51)	19% (12)	3.2% (2)	12.7% (8)
Yes	80% (36)	20% (9)	4.4% (2)	33.3% (15)
*p* value	0.902	0.902	> 0.999	0.010*
Smoking				
No	80% (56)	17.1% (12)	5.7% (4)	24.3% (17)
Yes	81.6% (31)	23.7% (9)	0% (0)	15.8% (6)
*p* value	0.843	0.412	0.295	0.303

*Significant at 5%

## Discussion

Despite a significant improvement in the management of HFrEF in recent years, it remains the major cause of morbidity and mortality in cardiac patients. With the favorable results from various clinical trials [10–12], Sacubitril/Valsartan is a class I indication in patients with HFrEF [7–9]. Nonetheless, due to its dual mode of action, its safety and tolerability along with optimal dosage and up-titration remained a major concern among the physicians. Additionally, effectiveness of Sacubitril/Valsartan in Asian HF patients is still a point of discussion as its benefits in the Asian sub-group of PARADIGM-HF trial fell short of reaching the statistical significance [13]. Therefore, in the current study our aim was to implement a systematic up-titration regime along with the assessment of safety and tolerability of Sacubitril/Valsartan for HFrEF patients in Pakistani population. After 12 weeks of observation, a majority 80.6% of the patients well tolerated the prescribed therapy. A significantly improved functional class was also witnessed with 75.0% in class I and 24.1% in class II, and only one (0.9%) patient in class III at the end of 12 weeks. Although, hyperkalemia (21.3%) remains the main safety concern followed by hypotension (19.4%), and renal dysfunction (3.7%) in our population.

In the PARADIGM-HF study hypotension was the leading safety concern among South Asian HF patients received Sacubitril/Valsartan with the incidence rate of 10.5% followed by hyperkalaemia in 8.9%, and angioedema in 0.3% [13]. Corresponding incidence rates in our study are higher than PARADIGM-HF possibly because of differences in population physical characteristics such as shorter structure and lower body weight, which is why comparatively lower doses of drugs are advocated for the Asians [14, 15]. Additionally, the incidence of hypotension was found to be more common among normotensive patients, female patients had higher risk of renal dysfunction, and hyperkalemia was found to be more common in diabetic patients. This finding could be possibly due to some extent of sub-clinical renal dysfunction. The PARALLEL-HF study reported safety and well tolerance of Sacubitril/Valsartan among Japanese patients with HFrEF compared to enalapril [16].

Findings of our study reading tolerability of Sacubitril/Valsartan in our population are reassuring with more than 80% tolerability similar to the TITRATION study where tolerability success was achieved in 85.2% of the patients treated with Sacubitril/Valsartan [11]. A maximum dose of 200 mg bid was selected in this study as this level of dose was well tolerated in both TITRATION and PARADIGM-HF studies [11, 13]. The TITRATION study evaluated gradual initiation/up-titration from 50 to 200 mg bid over three or six weeks [17]. Gradual up-titration regime over 6 weeks, compared to 3 weeks, was more effective in achieving and maintaining the target dose of Sacubitril/Valsartan. Therefore, in our study we adopted stepwise up-titration regime from initial does of 50 to the target dose of 200 mg bid over a 6 weeks period. The up-titration regime was in accordance with safety and tolerability of the patient with the dosage rather than forced up-titration. Among the non-tolerating patients we observed increased complains of vertigo, symptomatic, increased creatinine and potassium level, hence, a systematic titration based on patients physical as well as laboratory examination with close monitoring can improve the tolerability of Sacubitril/Valsartan in these patients. In real-life clinical practice, several patient related (such as race, age, or co-morbid conditions) as well as system related (availability or accessibility to the health care system) factors can influence the tolerability of Sacubitril/Valsartan among these patients. A study by Hsu et al. [18] reported the PREDICT-HF model to be a useful clinical model for risk stratification of patients with HFrEF. The permanent discontinuation of Sacubitril/Valsartan therapy as found to be 8.3 per 100 patient-years for high risk patients as compared to 2.5 per100 patient-years for patients with standard-risk. The commonest reported reason for discontinuation of Sacubitril/Valsartan therapy was hypotension (37.9%) followed by hyperkalemia or renal impairment (19.7%), and allergic reaction or adverse effects (13.6%) [18].

Some of the recent studies have demonstrated safety and efficacy Sacubitril/Valsartan in real-life study of patients with HFrEF. A study conducted by Armentaro et al. [19], the Sacubitril/Valsartan therapy in HFrEF patients was observed to improve NYHA functional class with the improvement of renal function, reduction of NT-proBNP levels, and improvement in several hemodynamic, clinical, and echocardiographic parameters during 2-year follow-up of 60 patients [19]. In another study Armentaro et al. [20] reported a potential therapeutical role of Sacubitril/Valsartan therapy in HFrEF patients with metabolic co-morbid conditions. In addition to functional and echocardiographic improvements, a persistent metabolic improvement has been observed over the follow-up period of 12 months [20].

Even though, this is the first prospective clinical study in Pakistani population, single center experience, lack of control group, and limited sample size remained the main limitation of our study. The short duration of follow-up remained another important limitation considering the chronic nature of the diseases. Additionally, in real-life clinical practice HFrEF patients generally present with multiple co-morbid conditions, hence, exclusion of low eGFR, low BP, low hemoglobin, and CRT/ICD treatment may limit the generalizability of study findings. Further large scale multicenter randomized studies with longer follow-up duration are needed to elaborate the role of Sacubitril/Valsartan in HFrEF patients of Pakistani population.

## Conclusion

In conclusion, Sacubitril/Valsartan therapy in HFrEF patients is safe and moderately tolerated among the Pakistani population with stable hemodynamic parameters and significant improvement in left ventricular function and function class. Hence, it can be used as first line of treatment for the patients with HFrEF with a gradual up-titration regime over a 6 weeks period.

## Data Availability

Data and material will be available upon request to the corresponding author.

## Abbreviations

ARNI: Angiotensin receptor blocker and a neprilysin inhibitor
HFrEF: Heart failure with reduced ejection fraction
NICVD: National Institute of Cardiovascular Diseases
HF: Heart failure
ACEIs: Angiotensin-converting enzyme inhibitors
ARB: Angiotensin receptor blocker
FDA: Food and Drug Administration
EMA: European Medicines Agency
ACC: American College of Cardiology
AHA: American Heart Association
ESC: European Society of Cardiology
NYHA: New York Heart Association
LVEF: Left ventricular ejection fraction
eGFR: Estimated glomerular filtration rate
